# Supplementary material for: Development of an amplicon-based sequencing approach in response to the global emergence of mpox
Source: PLoS Biol. 2023 Jun 13;21(6):e3002151. doi: 10.1371/journal.pbio.3002151 (PMC10263305; doi:10.1371/journal.pbio.3002151)
Supplement: S2 Table — Coverage at 46 clade- and lineage-defining positions [19] was determined based on 15 samples sequenced by the CDPH/YSPH with Ct <31 and down-sampled to 1 million sequencing reads. Positions are listed for the NCBI mpox reference genome (NC_063383) and the reference genome (MT903345) used in this study. (DOCX) [file pbio.3002151.s002.docx]

| **Clade/**  **Lineage** | **Position**  **(Ref: NC_063383)** | **Position**  **(Ref: MT903345.1)** | **Range of Coverage** | **# samples with ≥10X coverage** | **Amplicon(s)** |
| --- | --- | --- | --- | --- | --- |
| clade I/ clade II | 86502 | 86527 | 65 - 976 | 15/15 | 72, 73 |
| clade I/ clade II | 35352 | 35377 | 75 - 761 | 15/15 | 29 |
| clade II | 150970 | 150995 | 270 - 2085 | 15/15 | 124, 125 |
| clade IIa | 54013 | 54038 | 375 - 1364 | 15/15 | 44, 45 |
| clade IIb | 48148 | 48173 | 61 - 566 | 15/15 | 39, 40 |
| A | 48527 | 48552 | 42 - 405 | 15/15 | 39, 40 |
| A | 19367 | 19392 | 3 - 69 | 13/15 | 16 |
| A.1 | 83326 | 83351 | 83 - 962 | 15/15 | 69, 70 |
| A.1.1 | 34459 | 34484 | 1202 - 4733 | 15/15 | 28, 29 |
| A.2 | 34472 | 34497 | 1159 - 4516 | 15/15 | 28, 29 |
| A.2.1 | 140492 | 140517 | 0 - 37 | 12/15 | 116, 117 |
| A.2.1 | 25072 | 25097 | 2 - 259 | 11/15 | 21 |
| A.2.1 | 179537 | 179562 | 13 - 186 | 15/15 | 149 |
| A.2.2 | 103019 | 103044 | 5 - 52 | 13/15 | 86 |
| A.2.2 | 21991 | 22016 | 8 - 126 | 14/15 | 18 |
| A.2.2 | 158424 | 158449 | 159 - 1029 | 15/15 | 131, 132 |
| A.2.3 | 74226 | 74251 | 444 - 5247 | 15/15 | 61, 62 |
| A.2.3 | 57284 | 57309 | 41 - 212 | 15/15 | 47, 48 |
| A.3 | 100971 | 100996 | 0 - 22 | 7/15 | 84 |
| A.3 | 96841 | 96866 | 45 - 212 | 15/15 | 81 |
| B.1 | 77383 | 77408 | 448 - 2829 | 15/15 | 64, 65 |
| B.1.1 | 74360 | 74385 | 14 - 185 | 15/15 | 62 |
| B.1.10 | 89906 | 89931 | 0 - 11 | 1/15 | 75 |
| B.1.10 | 94798 | 94823 | 104 - 391 | 15/15 | 79 |
| B.1.11 | 159277 | 159302 | 526 - 2030 | 15/15 | 131, 132 |
| B.1.11 | 18133 | 18158 | 13 - 112 | 15/15 | 15 |
| B.1.12 | 182950 | 182975 | 942 - 2832 | 15/15 | 152, 153 |
| B.1.13 | 175093 | 175118 | 2 - 372 | 10/15 | 145 |
| B.1.14 | 159779 | 159804 | 3 - 55 | 12/15 | 132 |
| B.1.14 | 36617 | 36642 | 34 - 1090 | 15/15 | 30, 31 |
| B.1.15 | 151362 | 151387 | 121 - 1031 | 15/15 | 125 |
| B.1.15 | 149818 | 149843 | 867 - 3670 | 15/15 | 123, 124 |
| B.1.16 | 185557 | 185582 | 139 - 1364 | 15/15 | 154, 155 |
| B.1.16 | 161797 | 161822 | 415 - 5920 | 15/15 | 133, 134 |
| B.1.16 | 9389 | 9414 | 20 - 257 | 15/15 | 7, 8 |
| B.1.17 | 12169 | 12194 | 1 - 22 | 6/15 | 10 |
| B.1.17 | 44960 | 44985 | 34 - 164 | 15/15 | 37 |
| B.1.2 | 186165 | 186190 | 49 - 460 | 15/15 | 154, 155 |
| B.1.3 | 190660 | 190685 | 89 - 540 | 15/15 | 158, 159 |
| B.1.4 | 34308 | 34333 | 1546 - 6848 | 15/15 | 28, 29 |
| B.1.5 | 70780 | 70805 | 0 - 21 | 4/15 | 59 |
| B.1.6 | 111029 | 111054 | 529 - 3819 | 15/15 | 92, 93 |
| B.1.7 | 25644 | 25669 | 0 - 424 | 10/15 | 21, 22 |
| B.1.8 | 5595 | 5620 | 100 - 525 | 15/15 | 5 |
| B.1.8 | 191615 | 191640 | 353 - 3999 | 15/15 | 159, 160 |
| B.1.9 | 181367 | 181392 | 392 - 2479 | 15/15 | 150, 151 |
