# Supplementary figures and images for: Development of an amplicon-based sequencing approach in response to the global emergence of mpox
Source: PLoS Biol. 2023 Jun 13;21(6):e3002151. doi: 10.1371/journal.pbio.3002151 (PMC10263305; doi:10.1371/journal.pbio.3002151)

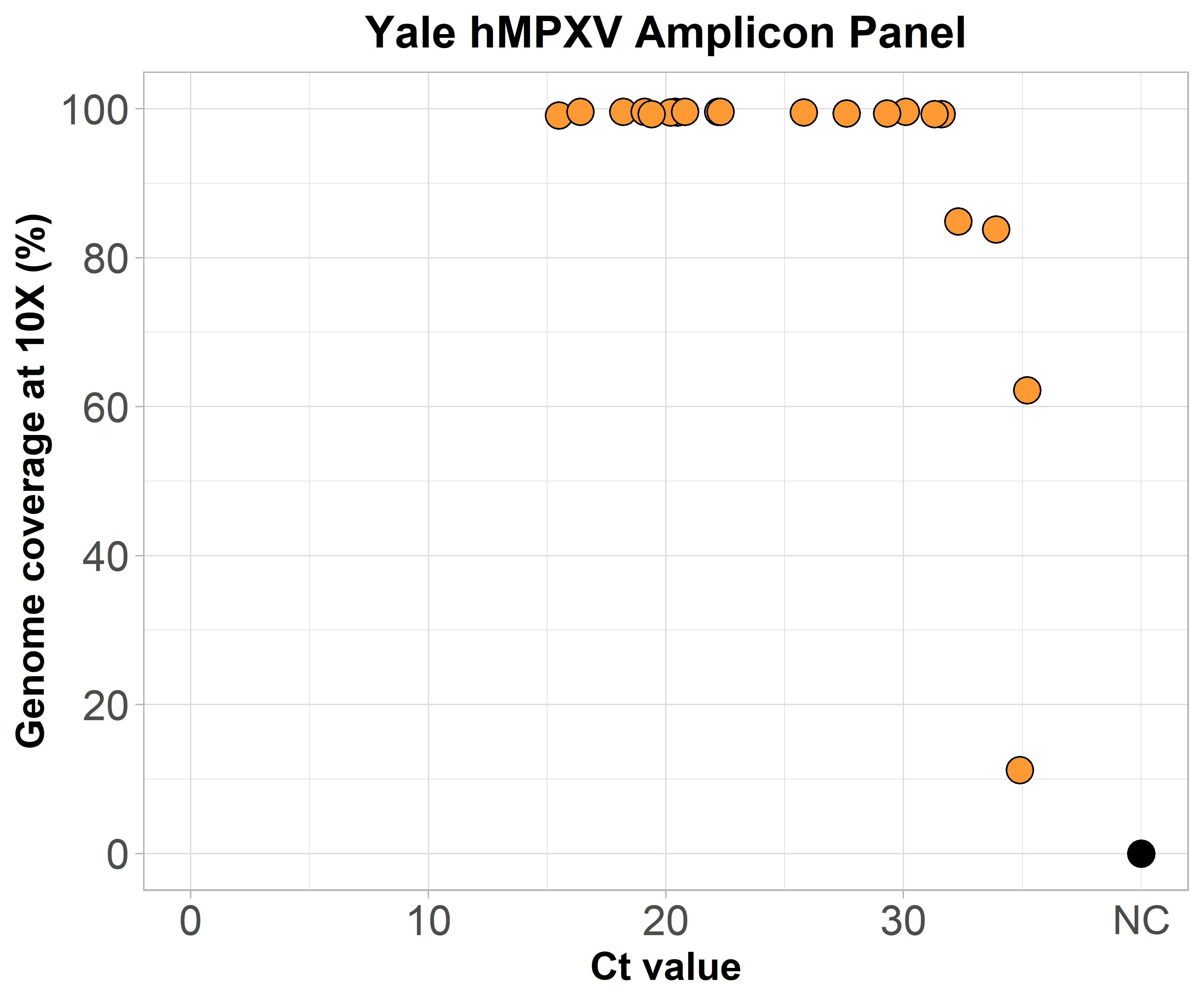

Supplement: S1 Fig — Lesion swabs (N = 21) were re-sequenced using the Yale hMPXV amplicon panel instead of manually pooled primers. NC = negative control. Source data can be found in S6 Data. (TIF) [file pbio.3002151.s004.tif]
