# Supplementary material for: Development of an amplicon-based sequencing approach in response to the global emergence of mpox
Source: PLoS Biol. 2023 Jun 13;21(6):e3002151. doi: 10.1371/journal.pbio.3002151 (PMC10263305; doi:10.1371/journal.pbio.3002151)
Supplement: Attachment — Submitted filename: Rebuttal.pdf [file pbio.3002151.s011.pdf]

## Reviewer #1

> The work described in the manuscript "Development of an amplicon-based sequencing approach in response to the global emergence of human monkeypox virus" by Chen et al represents a technical advance in whole genome sequencing amplification of a poxvirus genome using highly multiplexed amplicon-based sequencing. However, its importance is very difficult to assess since the manuscript lacks sufficient evidence that the high coverage achieved with this method actually covers areas of significant variability and phylogenetically informative in the context of the current outbreak. In simple words, it does not matter really if the coverage is high, if it actually does not cover the phylogenetically informative sites.

We thank the reviewer for taking the time to review our manuscript and provide feedback. We further investigated coverage at phylogenetically informative sites, and **have now added a supplementary table (Supplementary Table 2) comparing coverage for 15 of our samples to 46 lineage-defining genome sites, including 25 within the outbreak clade IIb**. We have additionally addressed this point of feedback in the results and discussion sections and note that none of the 46 sites have <10X coverage across all of our samples, indicating good coverage of phylogenetically informative sites.

***"To further understand how this variation may affect coverage at phylogenetically relevant sites, we compared the coverage of the same 15 samples with Ct <31 and down-sampled to 1 million reads/sample to 46 clade- and lineage-defining mutations [19]. We found that none of the 46 sites had a coverage of <10X across all samples and that only four sites, associated with the A.3, B1.1.10, B.1.17, and B.1.5 lineages, had <10X coverage for more than half of the samples (Supplementary Table 2). Additionally, we show that none of the previously identified amplicons with low mean coverage, with the exception of amplicon 59, are associated with low coverage for these phylogenetically informative sites. These findings suggest that with high quality samples and a sufficient number of reads per sample, this approach can consistently identify clade- and lineage-defining mutations."***

**Supplementary Table 2:** Depth of coverage at phylogenetically informative genome sites. Coverage at 46 clade- and lineage-defining positions [19] was determined based on 15 samples sequenced by the CDPH/YSPH with Ct <31 and down-sampled to 1 million sequencing reads. Positions are listed for the NCBI mpox reference genome (NC_063383) and the reference genome (MT903345) used in this study.

| Clade/ Lineage | Position (Ref: NC_063383) | Position (Ref: MT903345.1) | Range of Coverage | # samples with ≥10X coverage | Amplicon(s) |
|---|---|---|---|---|---|
| clade I/ clade II | 86502 | 86527 | 65 - 976 | 15/15 | 72, 73 |
| clade I/ clade II | 35352 | 35377 | 75 - 761 | 15/15 | 29 |
| clade II | 150970 | 150995 | 270 - 2085 | 15/15 | 124, 125 |
| clade IIa | 54013 | 54038 | 375 - 1364 | 15/15 | 44, 45 |
| clade IIb | 48148 | 48173 | 61 - 566 | 15/15 | 39, 40 |
| A | 48527 | 48552 | 42 - 405 | 15/15 | 39, 40 |
| A | 19367 | 19392 | 3 - 69 | 13/15 | 16 |
| A.1 | 83326 | 83351 | 83 - 962 | 15/15 | 69, 70 |
| A.1.1 | 34459 | 34484 | 1202 - 4733 | 15/15 | 28, 29 |

| | | | | | |
|---|---|---|---|---|---|
| A.2 | 34472 | 34497 | 1159 - 4516 | 15/15 | 28, 29 |
| A.2.1 | 140492 | 140517 | 0 - 37 | 12/15 | 116, 117 |
| A.2.1 | 25072 | 25097 | 2 - 259 | 11/15 | 21 |
| A.2.1 | 179537 | 179562 | 13 - 186 | 15/15 | 149 |
| A.2.2 | 103019 | 103044 | 5 - 52 | 13/15 | 86 |
| A.2.2 | 21991 | 22016 | 8 - 126 | 14/15 | 18 |
| A.2.2 | 158424 | 158449 | 159 - 1029 | 15/15 | 131, 132 |
| A.2.3 | 74226 | 74251 | 444 - 5247 | 15/15 | 61, 62 |
| A.2.3 | 57284 | 57309 | 41 - 212 | 15/15 | 47, 48 |
| A.3 | 100971 | 100996 | 0 - 22 | 7/15 | 84 |
| A.3 | 96841 | 96866 | 45 - 212 | 15/15 | 81 |
| B.1 | 77383 | 77408 | 448 - 2829 | 15/15 | 64, 65 |
| B.1.1 | 74360 | 74385 | 14 - 185 | 15/15 | 62 |
| B.1.10 | 89906 | 89931 | 0 - 11 | 1/15 | 75 |
| B.1.10 | 94798 | 94823 | 104 - 391 | 15/15 | 79 |
| B.1.11 | 159277 | 159302 | 526 - 2030 | 15/15 | 131, 132 |
| B.1.11 | 18133 | 18158 | 13 - 112 | 15/15 | 15 |
| B.1.12 | 182950 | 182975 | 942 - 2832 | 15/15 | 152, 153 |
| B.1.13 | 175093 | 175118 | 2 - 372 | 10/15 | 145 |
| B.1.14 | 159779 | 159804 | 3 - 55 | 12/15 | 132 |
| B.1.14 | 36617 | 36642 | 34 - 1090 | 15/15 | 30, 31 |
| B.1.15 | 151362 | 151387 | 121 - 1031 | 15/15 | 125 |
| B.1.15 | 149818 | 149843 | 867 - 3670 | 15/15 | 123, 124 |
| B.1.16 | 185557 | 185582 | 139 - 1364 | 15/15 | 154, 155 |
| B.1.16 | 161797 | 161822 | 415 - 5920 | 15/15 | 133, 134 |
| B.1.16 | 9389 | 9414 | 20 - 257 | 15/15 | 7, 8 |
| B.1.17 | 12169 | 12194 | 1 - 22 | 6/15 | 10 |
| B.1.17 | 44960 | 44985 | 34 - 164 | 15/15 | 37 |
| B.1.2 | 186165 | 186190 | 49 - 460 | 15/15 | 154, 155 |
| B.1.3 | 190660 | 190685 | 89 - 540 | 15/15 | 158, 159 |
| B.1.4 | 34308 | 34333 | 1546 - 6848 | 15/15 | 28, 29 |
| B.1.5 | 70780 | 70805 | 0 - 21 | 4/15 | 59 |
| B.1.6 | 111029 | 111054 | 529 - 3819 | 15/15 | 92, 93 |
| B.1.7 | 25644 | 25669 | 0 - 424 | 10/15 | 21, 22 |
| B.1.8 | 5595 | 5620 | 100 - 525 | 15/15 | 5 |
| B.1.8 | 191615 | 191640 | 353 - 3999 | 15/15 | 159, 160 |
| B.1.9 | 181367 | 181392 | 392 - 2479 | 15/15 | 150, 151 |

The authors emphasize that there is an urgent need for a sensitive and broadly applicable sequencing approach, but they do not provide enough data to show that the amplicon-based sequencing approach is sensitive enough to detect the variations that ARE important for understanding the transmission of the virus.

Without a priori knowledge of which genome sites could give rise to phenotypic changes related to virus transmission, it is not possible to definitively state the degree to which our scheme covers each of these sites. **However, we have clarified that our approach can be used to detect mutations/deletions that affect clinical diagnostics and therapeutics**. This impact on diagnostic and therapeutic efficacy has significant implications for population level transmission dynamics.

***"For instance, our method has already proven successful in identifying a 600 bp deletion that affects commonly used RT-PCR based clinical diagnostic assays [26,27]. Furthermore, our approach can be used to detect a mutation in the VP37 gene resulting***

***in resistance to Tecovirimat, an antiviral medication widely used in the treatment of mpox, further highlighting the value of regular genomic surveillance to detect clinically important mutations [29,30].”***

> While the amplicon-based sequencing approach described in the manuscript is clearly an improvement over shotgun sequencing for whole genome sequencing of monkeypox virus, there is no evidence presented that shows that the information generated is useful for handling the outbreak. While real-time molecular epidemiology is a revolutionary tool for understanding the transmission of infectious diseases, it is not clear that this approach can be applied to large DNA viruses such as mpox virus in an extremely short timeline. The authors obviously have that information, since their sampling is comprehensive from multiple parts of the world. So, it is somehow strange that the manuscript does not include any evaluation of the diversity described.

The focus of our manuscript is on the development and evaluation of the amplicon-based sequencing approach, and phylogenetic analyses were outside the scope of this study. Data generated with our approach have been used in other studies focused on phylogenetics of the monkeypox virus outbreak, and the detection of a deletion affecting clinical diagnostic assays. We have now added a discussion of the potential use cases of our approach in outbreak response, highlighting examples of how genomic surveillance can 1) inform public health surveillance, 2) monitor clinical diagnostic assays, and 3) detect mutations associated with resistance to antivirals. To this third point, with the same 15 samples used to generate Supplementary Table 2, our approach generates >10X coverage (range: 81-743) for the amino acid position (277) associated with resistance to Tecomirivat, an antiviral medication widely used in the treatment of mpox cases.

***“Public health surveillance has been implemented to understand factors that may have contributed to the rapid global spread of mpox in 2022 [28], including evidence for human adaptation driven by the APOBEC3 enzyme [12]. In outbreak settings, whole genome sequencing can be used to detect the introduction of new lineages, identify mutations associated with phenotypic adaptations, assess transmission dynamics and intervention effectiveness, and guide clinical decision making. For instance, our method has already proven successful in identifying a 600 bp deletion that affects commonly used RT-PCR based clinical diagnostic assays [26,27]. Furthermore, our approach can be used to detect a mutation in the VP37 gene resulting in resistance to Tecovirimat, an antiviral medication widely used in the treatment of mpox, further highlighting the value of regular genomic surveillance to detect clinically important mutations [29,30].”***

> Moreover, the authors do not provide any data on the accuracy and reproducibility of the method, which is crucial for evaluating its usefulness for public health surveillance efforts.
> Additionally, the authors do not provide evidence of how cross-reactive this system would be with other mpox or orthopoxvirus. Is there a risk that this system is too strain-specific? The authors only discuss this problem in the context of how easy would be to replace/complement the primer set with new additions to fix drop outs. But they do not address how likely this panel would be able to detect other lineage introductions.

We have clarified that our approach was independently implemented in each of our collaborating laboratories with similarly successful results, reinforcing the reproducibility of this method across a range of settings.

***"By collaborating with 10 public health labs across the world, we show that this approach can be independently and effectively implemented across a range of settings, experience levels, and resource demands."***

We have also clarified that our approach is not intended to be used as a diagnostic assay and is only intended for public health surveillance. As a result, cross-reactivity is not a concern. However, if other orthopoxviruses are present in a clinical sample and would be amplified, then their presence would be clear and easily differentiable from human monkeypox virus in the bioinformatics analysis.

***"The intended use for this amplicon-based sequencing approach is for public health surveillance and it is not intended as a diagnostic assay. As such, we did not evaluate cross-reactivity with other orthopoxviruses. Similarly to the genomic surveillance systems used for SARS-CoV-2, sequencing should be performed with confirmed positive cases to generate data used to guide public health responses. While we did not test this approach with other orthopoxviruses, if they were to be present in a sample and able to be partially amplified, then it would be evident in the bioinformatics analysis and could serve as an additional use case for this approach."***

We investigated coverage across 46 phylogenetically informative sites, including clade- and lineage-defining mutations outside the outbreak clade. Most sites had high coverage across all samples, and none of the sites had consistent low coverage <10. This shows that our approach is able to generate sufficient coverage for clade or lineage identification.

***"Moreover, we show that none of the sites associated with clade- and lineage- defining mutations have consistently low coverage."***

| |
|---|
| One of the key considerations for implementing a strain-specific sequencing system like the one described in the manuscript in low- and middle-income countries (LMICs) or endemic areas of Africa where MPXV is circulating is the availability of resources and infrastructure. While the authors highlight the cost-effectiveness of the amplicon-based sequencing approach, it is important to consider the broader economic and technological landscape of LMICs and endemic areas. In many LMICs and endemic areas, there may be limited resources for public health interventions, let alone advanced molecular sequencing technologies. But, even if the infrastructure were available, it is important to evaluate the return of investment of generating the data. |

We agree with the reviewer that this is an important consideration. We have clarified that our approach employs many of the same resources and infrastructure that were developed in LMICs for SARS-CoV-2 sequencing, such that this approach could be implemented with little to no additional demands on resources. We also highlight the overall rapid advancements and

investments made to genomic surveillance systems in LMICs (including those in endemic regions) as a result of the COVID-19 pandemic.

***"Notably, this approach could see widespread adoption amongst low- or middle-income countries (LMICs), many of which saw rapid expansion of their sequencing infrastructure during the COVID-19 pandemic [21,22]. Indeed, one of the few positive developments of the pandemic has been the implementation of standardized genomic surveillance systems in LMICs, evident by the number of SARS-CoV-2 genomes submitted to genome databases from countries that previously lacked the capability for large scale whole genome sequencing. By utilizing many of the same sequencing resources (e.g. reagents, equipment, and bioinformatics pipelines) developed for use with SARS-CoV-2, our approach could provide a streamlined approach for genomic surveillance to aid LMICs in capacities ranging from case confirmations to outbreak response while placing little to no additional demands on resources. In particular, the outsourcing of traditionally computationally intensive workflows via the Terra platform and the ease of updating both the primer scheme and bioinformatics pipelines with our approach removes some of the barriers typically seen with implementing genomic surveillance in low resource settings."***

> Whole-genome sequencing is a powerful tool that can provide a detailed understanding of the genomic features of the virus and its transmission dynamics. However, it is also a resource-intensive process that requires significant human and analytical resources. Therefore, it is important to evaluate whether the information generated by whole-genome sequencing of monkeypox virus is worth the investment of resources, especially in the context of limited resources during an outbreak response.

We agree that the role of genomic surveillance should be placed in the context of resource demands relative to what it can offer public health responses. We highlight that, in addition to the identification of mutations that affect clinical diagnostics or antiviral medication efficacy, genomic surveillance could be implemented to confirm clinical cases, elucidate disease etiology, identify lineage introductions, assess transmission dynamics and intervention efficacies, and guide clinical decision making. Further, because this approach utilizes much of the pre-existing sequencing infrastructure established for the COVID-19 pandemic, the further investment of resources to facilitate monkeypox virus sequencing would be minimal.

***"In outbreak settings, whole genome sequencing can be used to detect the introduction of new lineages, identify mutations associated with phenotypic adaptations, assess transmission dynamics and intervention effectiveness, and guide clinical decision making."***

> Furthermore, it is important to consider the context-specific nature of the MPXV outbreak. In many cases, outbreaks may be localized and may not require the same level of molecular surveillance as a pandemic situation. In these contexts, the cost-benefit analysis of implementing a strain-specific sequencing system would likely be very different than in a pandemic situation.

We agree with the reviewer that the decision to implement any genomic surveillance systems would warrant a cost-benefit analysis by individual public health authorities contingent on their priorities and resources. We do not advocate for prioritizing molecular surveillance over other public health programs necessarily, and agree that the value added by molecular surveillance of monkeypox virus is context dependent. However, by designing our approach to plug into existing workflows we minimize additional cost demands, thereby enabling monkeypox virus surveillance in situations where it may be otherwise cost-prohibitive using other sequencing approaches. Altogether, we advocate for greater global surveillance of small or localized outbreaks, because we cannot predict which ones will expand globally. Availability of early sequences are critical for establishing the genomic clock and identifying the number and source of introductions into new areas.

> Since the authors are presenting their work basically as an enabling technology assessment, all of these components of the cost-benefit analysis should be evaluated and discussed, beyond the actual technical achievement of developing a clearly improved test.

We thank the reviewer for this feedback and have clarified the theoretical as well as the proven value monkeypox sequencing can have on outbreak response. We also discuss the potential for our approach to be integrated into existing sequencing workflows to minimize additional resource demands. However, we feel that we cannot provide a reliable cost-benefit analysis as this is dependent on many different factors that are variable across laboratories in various parts of the world. Instead, we argue that labs that already have established amplicon sequencing workflows can easily expand their current sequencing portfolio without the need for additional investment in new reagents, equipment, or training.

***"Notably, this approach could see widespread adoption amongst low- or middle-income countries (LMICs), many of which saw rapid expansion of their sequencing infrastructure during the COVID-19 pandemic [21,22]. Indeed, one of the few positive developments of the pandemic has been the implementation of standardized genomic surveillance systems in LMICs, evident by the number of SARS-CoV-2 genomes submitted to genome databases from countries that previously lacked the capability for large scale whole genome sequencing. By utilizing many of the same sequencing resources (e.g. reagents, equipment, and bioinformatics pipelines) developed for use with SARS-CoV-2, our approach could provide a streamlined approach for genomic surveillance to aid LMICs in capacities ranging from case confirmations to outbreak response while placing little to no additional demands on resources. In particular, the outsourcing of traditionally computationally intensive workflows via the Terra platform and the ease of updating both the primer scheme and bioinformatics pipelines with our approach removes many of the barriers typically seen with implementing genomic surveillance in low resource settings."***

## Reviewer #2

> This revision looks to establish an economically feasible sequencing approach for MPox during the 2022 outbreak. Kudos for policy translation in the discussion points. I have but minor points.

We thank the reviewer for their positive feedback and for taking the time to review our manuscript.

> In the Discussion, it is recommended that periodic long-read metagenomics sequencing be used to identify potential rearrangements in MPox. However, is it possible that something in the amplicon sequencing might trigger this necessity rather than guessing (something like the S gene drop out in SARS-CoV-2)? Else, is the purpose of this method only for infrastructure and economic means?

We thank the reviewer for their questions. Results from amplicon sequencing could indeed trigger further investigation. If changes occur in primer binding sites, this could result in amplicon drop-out which then triggers further investigation. If changes occur outside of primer binding sites, then the amplicon approach can identify these changes. For example, we used the amplicon approach to detect a 600 bp deletion that affected the performance of clinical diagnostic assays. We have clarified this in the discussion:

***“For instance, our method has already proven successful in identifying a 600 bp deletion that affects commonly used RT-PCR based clinical diagnostic assays [26,27].”***

> Is it a better move to invest in metagenomics infrastructure and pipelines that are flexible and plug-and-play for pathogens in the future? If so, then some discussion point of equity and LMIC should be made.

We thank the reviewer for this suggestion and have further addressed the implementation of this approach in LMICs. We highlight that by utilizing much of the existing infrastructure established in LMICs for SARS-CoV-2 sequencing, our plug-and-play approach significantly reduces resource demands and provides a more streamlined approach to pathogen genomic surveillance. The development of flexible, scalable metagenomics approaches would certainly be a worthwhile investment, and we see the development and infrastructure for such methods as complementary to the approach we have presented.

***“Notably, this approach could see widespread adoption amongst low- or middle-income countries (LMICs), many of which saw rapid expansion of their sequencing infrastructure during the COVID-19 pandemic [21,22]. Indeed, one of the few positive developments of the pandemic has been the implementation of standardized genomic surveillance systems in LMICs, evident by the number of SARS-CoV-2 genomes submitted to genome databases from countries that previously lacked the capability for large scale whole genome sequencing. By utilizing many of the same sequencing resources (e.g. reagents, equipment, and bioinformatics pipelines) developed for use with SARS-CoV-2, our approach could provide a streamlined approach for genomic surveillance to aid LMICs in capacities ranging from case confirmations to outbreak response while placing little to no additional demands on resources.”***

Regarding the impact of the manuscript, this is contextual. Is the amplicon coverage supposed to be used for identification and diagnostics or for phylogenetic studies? What is the primary intended utility of this assay?

Thank you for these questions, we have clarified that this approach is intended to be used for genomic surveillance and is not intended to be used as a diagnostic assay. To assess the utility of our approach in phylogenetic analyses, we have added a supplementary table **(Supplementary Table 2)** comparing coverage for 15 of our samples to 46 lineage-defining genome sites, including 25 within the outbreak clade IIb. We show that no position has <10X coverage across all of our samples, indicating that the genomes generated by this approach have sufficient coverage for phylogenetic analyses. In a genomic surveillance capacity, we have added a discussion on both the proven utility of this approach as well as other applications.

***"The intended use for this amplicon-based sequencing approach is for public health surveillance and it is not intended as a diagnostic assay. As such, we did not evaluate cross-reactivity with other orthopoxviruses. Similarly to the genomic surveillance systems used for SARS-CoV-2, sequencing should be performed with confirmed positive cases to generate data used to guide public health responses. While we did not test this approach with other orthopoxviruses, if they were to be present in a sample and able to be partially amplified, then it would be evident in the bioinformatics analysis and could serve as an additional use case for this approach. Public health surveillance has been implemented to understand factors that may have contributed to the rapid global spread of monkeypox in 2022 [28], including evidence for human adaptation driven by the APOBEC3 enzyme [12]. In outbreak settings, whole genome sequencing can be used to detect the introduction of new lineages, identify mutations associated with phenotypic adaptations, assess transmission dynamics, and intervention effectiveness, and guide clinical decision making. For instance, our method has already proven successful in identifying a 600 bp deletion that affects commonly used RT-PCR based clinical diagnostic assays [26,27]. Furthermore, our approach can be used to detect a mutation in the VP37 gene resulting in resistance to Tecovirimat, an antiviral medication widely used in the treatment of mpox, further highlighting the value of regular genomic surveillance to detect clinically important mutations [29,30]."***

Further, the issue of cross reactivity with other orthopoxviruses is not addressed and should be addressed at least in the Discussion.

We have clarified that this approach is not intended to be used as a diagnostic assay and that if other orthopoxviruses are able to be amplified, it would be clear in the bioinformatic analysis.

***"The intended use for this amplicon-based sequencing approach is for public health surveillance and it is not intended as a diagnostic assay. As such, we did not evaluate cross-reactivity with other orthopoxviruses. Similarly to the genomic surveillance systems used for SARS-CoV-2, sequencing should be performed with confirmed positive***

***cases to generate data used to guide public health responses. While we did not test this approach with other orthopoxviruses, if they were to be present in a sample and able to be partially amplified, then it would be evident in the bioinformatics analysis and could serve as an additional use case for this approach."***

How do the authors propose this method could be used in a fulminant outbreak where mutations are possible? Would a gene drop out be immediately detectable or predicted by this method? How does it compare to current metagenomics identification of mutations?!

Our approach can be used to identify the introduction of new lineages with distinct phenotypic characteristics, similarly to that of the SARS-CoV-2 variants. Gene drop outs can be detected in two ways: 1) if the amplicon spans the gene then the deletion would be detected (see example of 600 bp deletion that affects clinical diagnostics), or 2) if the gene spans a primer binding site then this would result in drop out of the amplicon that would trigger further investigation.

***"For instance, our method has already proven successful in identifying a 600 bp deletion that affects commonly used RT-PCR based clinical diagnostic assays [26,27]."***

The further development of amplicon sequencing primer schemes is most effective when implemented within a continuous quality improvement framework. Similar to what the ARTIC network has done for SARS-CoV-2, iterative updates in primer schemes can be made in response to low-coverage areas or when amplicon drop-outs are detected. However, given the slower evolutionary rate as compared to single-stranded RNA viruses, these updates are likely not needed as frequently.

***"As a double-stranded DNA virus with genetic proof-reading mechanisms, the monkeypox virus has a comparatively slower evolutionary rate than single-stranded RNA viruses [25]. Less genetic diversity over time translates to fewer differences between the reference genome used to generate the primer scheme and genomes that are associated with the 2022 outbreak (clade IIb), decreasing the risk for amplicon drop-out. As a result, the primer scheme may not have to be updated as frequently as, for example, the primer scheme used to sequence SARS-CoV-2, but iterative updates can be released if needed [14]."***
